# A deep learning method to integrate extracelluar miRNA with mRNA for cancer studies

**DOI:** 10.1093/bioinformatics/btae653

**Published:** 2024-11-04

**Authors:** Tasbiraha Athaya, Xiaoman Li, Haiyan Hu

**Affiliations:** Department of Computer Science, University of Central Florida, 4000 Central Florida BLVD, Orlando, FL, 32816, United States; Burnett School of Biomedical Sciences, University of Central Florida, 4000 Central Florida BLVD, Orlando, FL, 32816, United States; Department of Computer Science, University of Central Florida, 4000 Central Florida BLVD, Orlando, FL, 32816, United States

## Abstract

**Motivation:**

Extracellular miRNAs (exmiRs) and intracellular mRNAs both can serve as promising biomarkers and therapeutic targets for various diseases. However, exmiR expression data is often noisy, and obtaining intracellular mRNA expression data usually involves intrusive procedures. To gain valuable insights into disease mechanisms, it is thus essential to improve the quality of exmiR expression data and develop noninvasive methods for assessing intracellular mRNA expression.

**Results:**

We developed CrossPred, a deep-learning multi-encoder model for the cross-prediction of exmiRs and mRNAs. Utilizing contrastive learning, we created a shared embedding space to integrate exmiRs and mRNAs. This shared embedding was then used to predict intracellular mRNA expression from noisy exmiR data and to predict exmiR expression from intracellular mRNA data. We evaluated CrossPred on three types of cancers and assessed its effectiveness in predicting the expression levels of exmiRs and mRNAs. CrossPred outperformed the baseline encoder-decoder model, exmiR or mRNA-based models, and variational autoencoder models. Moreover, the integration of exmiR and mRNA data uncovered important exmiRs and mRNAs associated with cancer. Our study offers new insights into the bidirectional relationship between mRNAs and exmiRs.

**Availability and implementation:**

The datasets and tool are available at https://doi.org/10.5281/zenodo.13891508.

## 1 Introduction

microRNAs (miRNAs) are ∼22 nucleotides long noncoding RNA that play important roles in gene regulation (Kim *et al.* 2009, Ding *et al.* 2018, Talukder *et al.* 2020). Extracellular miRNAs (exmiRs) are miRNAs found outside cells, circulating in various body fluids such as blood, urine, and saliva. They have been shown to play pivotal roles in facilitating intercellular communication (Happel *et al.* 2020).

High-throughput sequencing technologies have enabled comprehensive profiling of exmiR expression in a variety of body fluids across different cancer types (Yuan *et al.* 2016, Gilani *et al.* 2021). Analysis of exmiR expression data provides valuable information that contributes to early cancer diagnosis and the customization of therapeutic strategies, significantly benefiting cancer patients (Schwarzenbach *et al.* 2014). They have also been shown to be promising biomarkers for other diseases (Cortez *et al.* 2011). The noninvasive procedures of using body fluids as test samples eliminate the need for unnecessary repeat biopsies in diagnosing and monitoring the effectiveness of therapy (Happel *et al.* 2020). In addition, exmiR-based biomarkers offers advantages in terms of cost and assay simplicity (Schwarzenbach *et al.* 2014). However, current exmiR measurements are often noisy due to the issues of different exmiR profiling protocols and technical and biological variability (Carnino *et al.* 2019).

On the other hand, mRNA expression in tumor tissues has often been used for assessing cancer phenotypes (Mansoori *et al.* 2019). Various mRNA signatures have been identified for cancer prognosis (Ding *et al.* 2013, Robles *et al.* 2015, Wang *et al.* 2017, 2020, Ontario Health 2020, Ma *et al.* 2021). However, acquiring tumor mRNA data often requires invasive procedures and is more costly than obtaining exmiRs, which limits the use of intracellular mRNA (Janjic *et al.* 2022).

Studies have shown that integrating miRNA and mRNA expression profiles can better characterize phenotypes (Li *et al.* 2021, Talukder *et al.* 2022). To capitalize on the high-quality mRNA data while taking advantage of the lower cost of exmiR data, we introduce a deep learning encoder model named CrossPred. This model integrates mRNA and exmiR data from training samples to enhance the accuracy of predicting mRNA and exmiR expression in testing samples. In addition, CrossPred is designed to classify samples as cancerous or healthy based on the encoder output. To our knowledge, this is the first study to explore the combined use of mRNA and exmiR expression data for both cross-prediction of expression and cancer classification. We applied CrossPred to three types of cancer and demonstrated its effectiveness in predicting mRNA and exmiR expression as well as in classifying cancer samples.

The paper is organized as follows: Section 2 details the data and the CrossPred model. Section 3 presents the results. Finally, Section 4 discusses our findings.

## 2 Material and Methods

### 2.1 mRNA data

We obtained mRNA expression data for 603 lung, 1231 breast, and 446 gastric cancer samples from The Cancer Genome Atlas repository. Each sample involves 19 914 genes. Only genes with fewer than one percent zero expression values in all samples were kept for further analysis.

The mRNA expression data for 581, 459, and 359 healthy controls from lung, breast mammary, and stomach tissue, respectively, were downloaded from the Genotype-Tissue Expression (Lonsdale *et al.* 2013). Each sample contains 54 393 unique protein-coding genes, with mRNA expression in the form of read counts. Similarly, only genes with less than one percent zero expression values across all samples were retained for further usage.

The common genes present in both cancer and healthy controls were collected, totaling 13 271, 13 003, and 13 119 for lung, breast mammary, and stomach tissues, respectively. DESeq2 (Love *et al.* 2014) was used to identify the differentially expressed genes between cancer and healthy controls. Differentially expressed mRNAs with *P*-value <0.01 and a log 2-fold change >1 were selected for RPKM calculation. The resulting expression matrix was then used in the deep learning model.

### 2.2 exmiR data

We used the GSE106817 dataset for exmiR expression data. This dataset contains 115 individuals diagnosed with each cancer type (lung, breast, and gastric), with 2550 exmiRs’ expression profiled for each subject.

exmiR expression values were quantified by subtracting the background median from the spot median, and the dataset was preprocessed to eliminate low-quality data (Yokoi *et al.* 2018). To ensure balanced number of cancer and healthy controls, 115 subjects were randomly selected from the healthy controls. Next, the Limma package of R (Ritchie *et al.* 2015) was used to identify differentially expressed exmiRs with *P*-values smaller than 0.01 and log 2-fold change >1.

### 2.3 CrossPred

**Figure 1.**
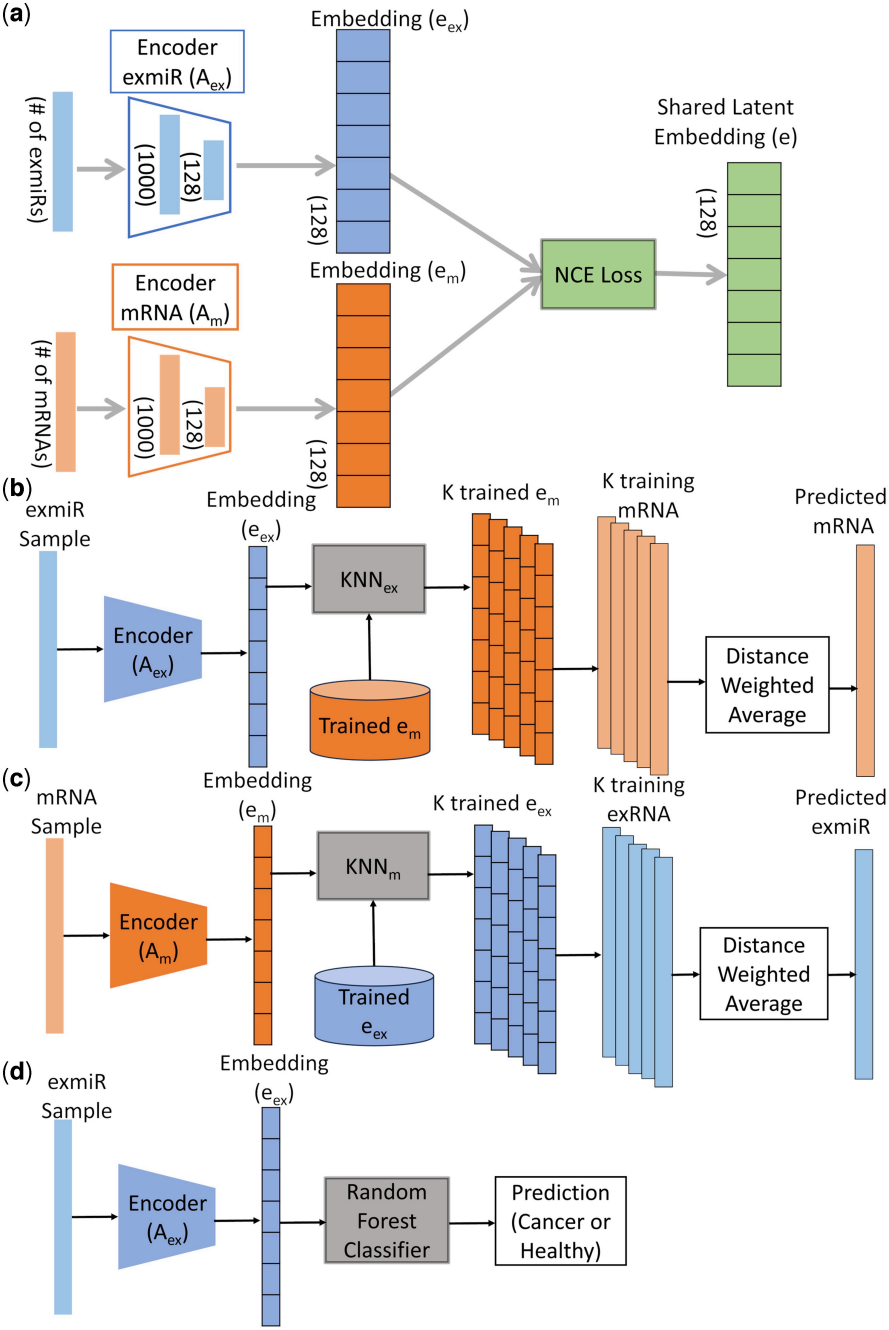
The CrossPred architecture. (a) Encoder model; (b) exmiR to mRNA prediction; (c) mRNA to exmiR prediction; (d) cancer prediction using exmiR expressions and $A_{ex}$ encoder. Using mRNA expression and $A_{m}$ encoder is similar.

CrossPred is a deep learning model that uses two encoders to integrate exmiR with mRNA data and creates a shared latent embedding. A K-nearest neighbors (KNN) model is then applied for data conversion based on the embedding space, facilitating the cross-prediction of expression and identification of important mRNAs and exmiRs relevant to cancer characterization. Furthermore, the shared embedding is also used for cancer sample classification (Fig. 1).

### 2.4 Training data preparation

We partitioned the data in Sections 2.1 and 2.2 with 60% for training, 5% for evaluation, and 35% for testing. The partition ratio was somewhat arbitrary, aiming to challenge the model with fewer training data and more testing data. To augment mRNA and exmiR input data for training, we devised a sampling method inspired by a upsampling technique (Chawla *et al.* 2002) to generate ten thousand cancerous sample pairs and ten thousand healthy sample pairs. We randomly selected one cancerous mRNA sample and paired it with one randomly selected cancerous exmiR sample to form a cancerous sample pair. Similarly, we randomly chose a healthy mRNA sample and paired it with a healthy exmiR sample to form a healthy sample pair. To avoid overfitting, we added Gaussian noise within the range of zero to the product of a small constant and one standard deviation of the expression values for every gene in each sample pair. This method preserved the integrity of the samples while inducing slight variations, enhancing the models’ generalization ability and mitigating the risk of overfitting.

### 2.5 Encoder model

The encoder model (Fig. 1a) integrates mRNA and miRNA data and produce a shared embedding space. It comprises two independent encoders: the $A_{m}$ encoder and the $A_{ex}$ encoder for mRNA and exmiR data, respectively. Both encoders share the same structure and design, capable of processing inputs from different data types with distinct features. Each encoder consists of three fully connected neural network layers (comprising 1000, 128, and 128 consecutive nodes, respectively), each of which is coupled with a batch normalization layer, followed by a ReLu activation function. The $A_{m}$ encoder and the $A_{ex}$ encoder generate embedding $e_{m}$ and embedding $e_{ex}$, respectively. These embeddings undergo a unique noise contrastive estimation (NCE) process and create a low dimensional shared latent space e. For training the encoders, the model uses an Adam optimizer with a batch size of 64, a learning rate of $5e^{-5}$, and 100 epochs. Notably, the training is fully unsupervised, facilitating the extraction of meaningful features from the data without the need for labeled samples.

### 2.6 The NCE loss function

The encoder embeddings $e_{m}$ and $e_{ex}\;$ contain information about the training mRNA and exmiR samples, respectively. The goal is to enhance similarity between mRNA and exmiR from the same category (cancerous or healthy. For instance, cancer mRNA and cancer exmiR or healthy mRNA and healthy exmiR are from the same category) and dissimilarity between mRNA and exmiR from different categories within the shared embedding space *e*. To do that, we need to maximize the mutual information of the embeddings ($e_{m}$ and $e_{ex})$ to project them into a shared embedding space *e*. Contrastive learning is a method for maximizing such mutual information, typically involving the pairing of samples. In our case, the mRNA-exmiR pair from the same sample category (cancerous or healthy) is considered a positive pair, while the sample pairs from different categories are deemed negative pairs (Amid and Warmuth 2019). Our implementation adopted a unique NCE loss-based training approach for contrastive learning (Xu *et al.* 2022) (Section 2.4). The NCE was applied to the embedding space ($e_{m}$ and $e_{ex}$) to create a shared latent space *e* capable of effectively distinguishing between cancer and healthy samples using mRNA and exmiR data. The NCE loss (L) is given below:

$$L_{m,ex}=-\log\frac{e^{\frac{s(e_{m}^{i},e_{ex}^{i})}{\tau }}}{\sum_{j=1(j\neq i)}^{n}e^{\frac{s(e_{m}^{i},e_{ex}^{j})}{\tau }}}$$

Here, i and j are the positive sample pair ID, n is the total number of sample pairs in a batch of data (*n* = 64), the similarity function *s* between the $e_{m}$ and $e_{ex}$ embeddings is a dot product of the two vectors, and τ is the temperature parameter. The similarity score would be high for mRNA and exmiR pairs from the same category. Conversely, the similarity score would be lower for mRNA and exmiR pairs from different categories. This distinction in similarity scores allows the model to effectively differentiate between samples of two categories based on their mRNA and exmiR profiles.

The training, validation, and test datasets were kept entirely separated throughout the process. During training, we continuously evaluated the model using the evaluation metrics in the results section with the validation dataset. Training stops when the overall score of the validation accuracy reaches a satisfactory level. Early stopping criteria were applied, terminating training when the sum of Pearson’s correlation and Spearman’s correlation ≥1.8 for both mRNA and exmiR validation data. We trained, validated, and tested th three cancer types separately.

### 2.7 KNN for cross-prediction

Following the training of the encoder networks, two KNN (${\mathrm{KNN}}_{m}$ and ${\mathrm{KNN}}_{ex}$) models were used for mRNA and exmiR predictions, respectively. K was set to 10 empirically after testing *K* = 5, 10, 15, 20, and 50. During the cross-prediction, the KNN model identifies the K nearest neighbors by computing the cosine distance between the embedding of the training data and the embedding of the testing data. The shared embedding space, learned through contrastive learning, enables KNN to leverage both mRNA and exmiR training data embeddings when calculating distances. In addition, KNN returns the indices of the nearest neighbors in the actual training data, enabling the calculation of a distance-weighted average of those data points to obtain the value of the desired predictions. For instance, when predicting mRNA from exmiR (Fig. 1b), KNN identifies the 10 nearest neighbors by computing the distance between the mRNA embeddings ($e_{m}$) of the training set and the test exmiR embedding. Next, the indices of 10 nearest neighbors of $e_{m}$ in the training set are retrieved. The predicted mRNA sample value is then obtained as the cosine distance-weighted average of these actual training mRNA data points. The formula to predict mRNA ($m_{\mathrm{pred}}$) using distance weighted average is given below.

$$m_{\mathrm{pred}}=\frac{\sum_{i=1}^{10}\frac{tr_{m}\left[I_{i}\right]}{d_{i}}}{\sum_{i=1}^{10}\frac{1}{d_{i}}},\;d_{i}={d^{\prime }}_{i}\;+\;e^{-10}$$

Here, d′ is the distance of the nearest neighbors and $tr_{m}$ is the training mRNA dataset and I is the index of the samples of the nearest neighbors. The same process is for mRNA to exmiR prediction (Fig. 1c).

### 2.8 Comparison with alternative models

We compared CrossPred with two alternative models: a common encoder-decoder (ED) and variational auto-encoder (VAE). Both alternatives are well-known Sequence-to-Sequence model capable of processing sequential data as input and generating sequential data as output (Talukder *et al.* 2021, Athaya *et al.* 2023). The ED model learns a deterministic, fixed latent representation to reconstruct inputs, focusing solely on minimizing reconstruction loss, while the encoder of a VAE produces parameters that describe a specific distribution in the latent space for each input. The VAE then enforces a Kullback-Leibler divergence term that requires this latent distribution to adhere to a multivariate Gaussian distribution, which ensures that the generated samples are coherent and consistent, facilitating better generation and interpolation of data. In brief, we design two separate ED models with identical structure, one for exmiR to mRNA prediction and the other for mRNA to exmiR prediction. The encoder comprises six linear layers coupled with a ReLU activation layer. These layers consist of 512, 256, 128, 64, 36, and 18 consecutive nodes, respectively. The decoder is symmetrically structured with six linear layers, each coupled with a ReLU activation function except for the last layer, where the decoder unit incorporates a sigmoid function. We use the mean squared error (MSE) loss function with the Adam optimizer in these models. VAE uses the same structure as the ED model. However, instead of producing a single deterministic latent vector, the encoder outputs the mean and variance of a multivariate Gaussian distribution, and the decoder then samples from this distribution to reconstruct the input. Similar to the CrossPred model, the ED and VAE models were trained using randomly paired training samples. For exmiR to mRNA prediction, randomly paired exmiR data serves as input for mRNA prediction, and vice versa for mRNA to exmiR prediction during training. However, since both models operate on a single data type as input to predict the other data type, it cannot leverage information from both types simultaneously to aid the predictions.

## 3. Results

### 3.1 Overall evaluation

To evaluate the models, we calculated the Pearson’s correlation coefficient between the mean expression of every gene across all samples of a specific cancer type and the mean predicted expression of this gene across these samples for all genes. We also measured the mean absolute error (MAE) of the predcited and actual expression of a gene in all samples of a particular cancer type. In all datasets, CrossPred had higher correlations, lower *P*-values and lower MAE than alternative models (Table 1). For instance, when predicting mRNA expression from exmiR expression in lung cancer, CrossPred had a correlation of 0.978, *P*-value 0, and MAE 0.883, while ED had 0.223, $3.45\times 10^{-23}$, and 5.566, and VAE had 0.247, $6.58\times 10^{-15}$, and 5.544, respectively (Table 1). CrossPred's superior performance compared with other models likely stems from its distinct training approach. While ED and VAE are trained on one type of data—either mRNA or exmiR—to predict the other, CrossPred combines both data types during training to create a shared embedding space. This allows it to leverage information from both mRNA and exmiR simultaneously, enhancing its predictive capabilities. In contrast, ED and VAE only utilize information from a single data type, which may explain why CrossPred outperforms them.

**Table 1.** Evaluation result for cross-prediction.

Prediction	Samples (cancer type)	Models	Pearson’s correlation	*P*-value of Pearson’s correlation	MAE
exmiR to mRNA	Lung	CrossPred	0.978	0	0.863
		ED	0.223	3.45E-23	5.566
		VAE	0.247	6.98E-15	5.554
	Breast	CrossPred	0.944	0	1.346
		ED	0.229	1.89E-24	5.318
		VAE	0.223	1.23E-22	5.315
	Gastric	CrossPred	0.963	0	0.718
		ED	0.278	1.23E-30	3.757
		VAE	0.249	1.02E-24	3.710
mRNA to exmiR	Lung	CrossPred	0.974	0	10.357
		ED	0.204	2.83E-25	25.014
		VAE	0.059	0.003	24.973
	Breast	CrossPred	0.985	0	5.479
		ED	0.161	1.20E-15	28.316
		VAE	0.065	0.001	28.313
	Gastric	CrossPred	0.946	0	8.011
		ED	0.042	0.033	23.588
		VAE	−0.097	9.02E-7	23.585

### 3.2 Prediction result for mRNA

#### 3.2.1 Differentially expressed mRNA prediction

**Figure 2.**
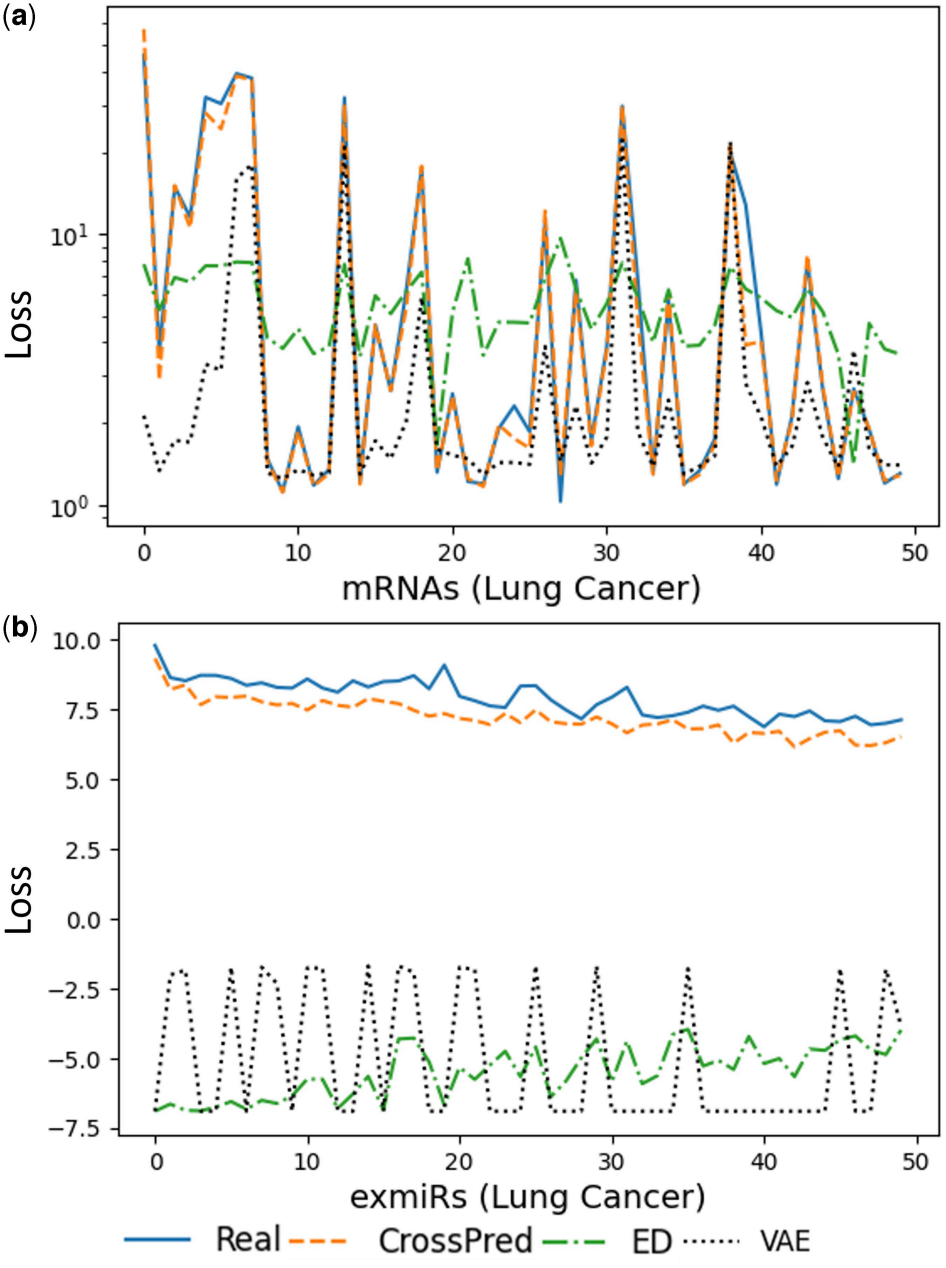
NLL of top 50 differentially expressed (a) mRNAs of lung cancer; (b) exmiRs of lung cancer. The *x*-axis of the figure denotes mRNA/exmiR index and y-axis denotes NLL.

To further compare the models, we focused on the top 50 differentially expressed mRNAs in each cancer. We modeled the expression of each mRNA by a negative binomial distribution as previously (Ren and Kuan 2020), for the actual and predicted expression across samples, respectively. Then we measured the negative loglikelihood loss (NLL) (Wu *et al.* 2021) of the predicted mRNA expression generated by the models (Fig. 2a and Supplementary Figs S1–S3). Remarkably, the fitted NLL curve for CrossPred predicted expression closely overlapped with the fitted NLL curve for the actual expression, which was far from the curves by alternative models.

#### 3.2.2 Important exmiRs for mRNA prediction

We investigated the significance of individual exmiRs in predicting mRNA expression. To identify important exmiRs for mRNA prediction, we introduced Gaussian noise into the input expression of each exmiR. Subsequently, we calculated the Euclidean distance between the predicted and actual mRNA gene expressions. The exmiR that are responsible for generating the highest distance was deemed the most crucial for predicting mRNA expression.

In lung cancer, the top five most important exmiRs for mRNA prediction were hsa-miR-4787-5p, hsa-miR-8069, hsa-miR-3665, hsa-miR-4488, and hsa-miR-3960. These exmiRs are actually playing potential roles in lung cancer pathogenesis and treatment. For instance, hsa-miR-4787-5p and hsa-miR-3665 have been implicated in resistance against epidermal growth factor receptor inhibitors, a common therapeutic approach in nonsmall cell lung cancer (Pal *et al.* 2021). hsa-miR-4488 is a biomarker for interstitial lung disease (Zhong *et al.* 2021).

In breast cancer, the top five most important exmiRs were hsa-miR-3960, hsa-miR-4488, hsa-miR-3665, hsa-miR-6869-5p, and hsa-miR-6729-5p. These exmiRs have also been implicated in breast cancer pathogenesis and diagnosis. For instance, hsa-miR-3960 exhibits increased expression levels in breast cancer tissues, suggesting its role as a diagnostic or prognostic biomarker (Zheng *et al.* 2020). hsa-miR-4488, detected within extracellular vesicles released by breast cancer cells, has shown promise in suppressing the angiogenesis of vascular endothelial cells (Dong *et al.* 2021), highlighting its therapeutic potential. has-mir-3665 is identified as a component in breast cancer detection methods and the development of breast cancer detection kits, underscoring its significance in early diagnosis and screening (Kondou *et al.* 2020). In addition, hsa-miR-6869-5p and has-mir-6729-5p are markers for breast carcinoma and have been incorporated into breast carcinoma detection devices (Kondou *et al.* 2024).

In gastric cancer, the top five most important exmiRs were hsa-miR-4488, hsa-miR-6869-5p, hsa-miR-8072, hsa-miR-3960, and hsa-miR-6729-5p. These exmiRs have been associated with various aspects of gastric cancer pathogenesis and diagnosis. For instance, hsa-miR-6729-5p is a potential biomarker for gastric cancer (Kozono *et al.* 2024). Similarly, has-miR-3960 is differentially expressed between the gastric cancer stem cells and their parental counterparts, indicating its potential role in tumor initiation and progression (Liu *et al.* 2014).

### 3.3 Prediction result for exmiR

#### 3.3.1 Differentially expressed exmiR prediction

Our exmiR data were from microarray experiments. The microarray expression data is commonly assumed to follow a Gaussian distribution, whether symmetric or skewed (Casellas and Varona 2012). We thus applied a Gaussian NLL (Nix and Weigend 1994) to model the expression of each of the top 50 differentially expressed exmiRs, for the predicted and actual expression, respectively. Similarly, the fitted Gaussian NLL for the predicted expression by CrossPred closely overlapped with the fitted Gaussian curve for the actual expression, which was obviously deviated from the curves modeled with expression predicted by alternative models (Fig. 2b and Supplementary Figs S4–S6).

#### 3.3.2 Important mRNAs for exmiR prediction

We identified the most important mRNAs to predict exmiR. In lung cancer, the five most important mRNAs were HBB, HBA2, MT2A, ZFP36, and DUSP1. Consistent induction of HBB has been observed in single-cell RNA-Seq profiles of circulating tumor cells from breast and lung cancers, suggesting its potential role as a biomarker (Zheng *et al.* 2017). Both HBB and HBA2 are shown to potentially impact the progression of nonsmall cell lung cancer (Kang *et al.* 2022). High expression of MT2A is associated with poor prognosis of lung adenocarcinoma (Yao *et al.* 2021). Upregulation of MT2A is also associated with poor survival in patients with nonsmall-cell lung cancer (Werynska *et al.* 2013). Loss of ZFP36 can promote proliferation, migration, and invasion of nonsmall cell lung cancer cells (Zhang *et al.* 2023). In addition, increased expression of DUSP1 has been implicated in lung cancer progression (Shen *et al.* 2016).

In breast cancer datasets, the five most important mRNAs were GABARAP, PFDN5, RPL21, CFB, and PCED1A. GABARAP could enhance the precision of both diagnosis and treatment approaches for breast cancer (Liu *et al.* 2021). Decreased levels of PFDN5 is observed in breast carcinoma specimens, indicating its potential role as a diagnostic or prognostic marker (Herranz-Montoya *et al.* 2021). In addition, CFB has emerged as a prognostic marker in breast cancer, with elevated expression being associated with a favorable prognosis (Lei *et al.* 2024).

In gastric cancer datasets, MT2A, TXNIP, FOS, RHOB, and GLUL emerged as the five most important mRNAs to predict exmiRs. TXNIP has a significantly reduced expression in gastric cancer tissues compared with normal tissues (Pan *et al.* 2022). The absence of FOS expression is associated with advanced stage, lymph node metastasis, lymphatic invasion, and shorter survival, indicating its loss during gastric cancer progression and its correlation with poor prognosis (Jin *et al.* 2007). In addition, RHOB expression significantly suppresses the proliferation, migration, and invasion of gastric cancer cells while also increasing their sensitivity to anticancer drugs (Zhou *et al.* 2011).

### 3.4 Cancer sample classification

Since CrossPred can reliably predict exmiR expression from mRNA expression and vice versa, we further studied whether the encoder output could distinguish cancer samples from healthy samples for each of the three cancer using exmiR or mRNA expressions as input (Fig. 1d). We found that the CrossPred reliably classified cancer samples and showed comparable or superior performance compared with four tools for cancer classification (Supplementary Tables S1 and S2).

We also evaluated the advantage of integrating exmiR data with miRNA data over using either exmiR data or mRNA data for cancer sample classification. We trained two additional models using mRNA-mRNA and exmiR-exmiR pairs as input, respectively. For the mRNA-mRNA model, which utilize only mRNA data, we used two nonoverlapping groups of mRNA data randomly divided from the original training mRNA data as input. The mRNA-mRNA model was then trained using the same NCE loss utilized in the CrossPred model. The exmiR-exmiR model was similarly trained. Subsequently, for each of the three models (CrossPred, mRNA-mRNA model, and exmiR-exmiR model), we computed the pairwise distance between a cancer sample point and a healthy sample point in the shared embedding space. A higher distance indicates superior model generalization and classification ability. Our analysis revealed that CrossPred consistently exhibited the largest separation distance between cancer and healthy samples across all evaluated scenarios (Supplementary Table S3), suggesting that integrating exmiR and mRNA strengthens our ability to distinguish cancer sampels from healthy ones.

## 4 Discussion

We developed the CrossPred method for bidirectional prediction of exmiR and mRNA expression levels. We demonstrated the good performance of CrossPred in predicting exmiR and mRNA expressions. Currently, no research has done the bidirectional prediction of exmiR and mRNA expressions. Most studies (Gilani *et al.* 2021, Sathipati *et al.* 2024) identify important features for cancer sample classification. CrossPred can also classify cancer sample without the hassle of feature selection.

It is worth mentioning that for each cancer type, the target mRNAs of the top differentially expressed exmiRs between cancer and healthy samples were significantly enriched in the top differentially expressed mRNAs (Supplementary Table S4). For instance, the targets of the top five exmiRs in lung cancer, breast cancer, gastric cancer are significantly enriched in the top 100 differentially expressed mRNAs for the specific cancer type, with the enrichment *P*-value as 0.008776402, 0.01470641, and 0.01827295 in lung, breast, and gastric cancer, respectively.

Despite the promising prospects of understanding the complex relationships between exmiRs and mRNAs in cancer studies, our work faces several limitations and challenges. For instance, the current sample size for exmiR expression is relatively small compared to the available mRNA data. Increasing the amount of exmiR data in the future would enable a more generalized model and provide more reliable results. In addition, the current exmiR data contains significant noise due to factors such as inconsistent profiling protocols and technical and biological variability (Carnino *et al.* 2019). Standardized protocols, like those being developed by the Extracellular RNA Communication Consortium's extracellular RNA atlas, will help refine the data quality. Moreover, our process for mRNA and exmiR prediction requires storing the training dataset with embedding data for the KNN model, which demands additional memory space. Developing a more memory-efficient model in the future will address this issue.

In summary, while more rigorous studies are needed to utilize exmiRs as clinical tools, our work has expanded the understanding of exmiRs and mRNAs by integrating them. The conversion between mRNA and exmiR expressions opens new avenues for innovative research approaches that bridge basic science with clinical applications.

## Supplementary Material

btae653_Supplementary_Data

## Data Availability

All data underlying this article are available in the article.
