# Supplementary material for: A deep learning method to integrate extracelluar miRNA with mRNA for cancer studies
Source: Bioinformatics. 2024 Nov 4;40(11):btae653. doi: 10.1093/bioinformatics/btae653 (PMC11565234; doi:10.1093/bioinformatics/btae653)
Supplement: btae653_Supplementary_Data [file btae653_supplementary_data.docx]

A Deep Learning Method to Integrate extracelluar miRNA with mRNA for cancer studies

Tasbiraha Athaya^1^, Xiaoman Li^2^, Haiyan Hu^1^

^1^Department of Computer Science, University of Central Florida, 4328 Scorpius Street, Florida, 32816, USA.

^2^Burnett School of Biomedical Science, College of Medicine, University of Central Florida, Orlando, Florida, United States of America.

We compared CrossPred with methods on cancer/normal sample classification. We first compared the performance of CrossPred with methods on cancer/normal sample classification. We selected two state-of-the art papers for each data type (mRNA and exmiR). The state-of-the-art papers were selected based on their reported prediction accuracy, dataset used, applied tools, and citations. If there were no paper that used data from all three cancer types in the same paper, we selected one of the cancer types for comparison. For example, we could not find any state-of-the-art paper that predicted all three cancer types using exmiR data with machine learning. So, we selected two recent papers that predicted breast cancer and gastric cancer using machine learning with exmiR data and reported high accuracy.

We implemented the tools using our preprocessed dataset and the evaluation metrics obtained are listed in the Table S1 and Table S2 below.

Supplementary Table S1. Evaluation result for Cancer Prediction with mRNA data.

| **Model** | **mRNAs**  **(Cancer type)** | **Accuracy (%)** | **Sensitivity (%)** | **Specificity (%)** | **F1 Score (%)** |
| --- | --- | --- | --- | --- | --- |
| CrossPred | Lung | 99.76 | 100 | 99.52 | 99.75 |
|  | Breast | 100 | 100 | 100 | 100 |
|  | Gastric | 100 | 100 | 100 | 100 |
| Ensemble  (Xiao *et al.*, 2018) | Lung | 99.27 | 98.52 | 100 | 99.26 |
|  | Breast | 99.38 | 98.76 | 100 | 99.38 |
|  | Gastric | 100 | 100 | 100 | 100 |
| WGAN and SVM  (Xiao *et al.*, 2021) | Lung | 50.84 | 0 | 100 | 0 |
|  | Breast | 50 | 0 | 100 | 0 |
|  | Gastric | 55.48 | 0 | 100 | 0 |

Supplementary Table S2. Evaluation result for Cancer Prediction with exmiR data.

| Model | exmiRs  (Cancer type) | Accuracy (%) | Sensitivity (%) | Specificity (%) | F1 Score |
| --- | --- | --- | --- | --- | --- |
| CrossPred | Lung | 97.56 | 95.12 | 100 | 97.50 |
|  | Breast | 100 | 100 | 100 | 1 |
|  | Gastric | 97.56 | 100 | 95.12 | 97.61 |
| XGboost  (Gilani *et al.*, 2022) | Gastric | 51.06 | 58.33 | 43.48 | 54.90 |
| BSig  (Sathipati *et al.*, 2024) | Breast | 50 | 100 | 0 | 66.67 |

References

Gilani,N. *et al.* (2022) Identifying Potential miRNA Biomarkers for Gastric Cancer Diagnosis Using Machine Learning Variable Selection Approach. *Frontiers in Genetics*, **12**.

Sathipati,S.Y. *et al.* (2024) An evolutionary learning-based method for identifying a circulating miRNA signature for breast cancer diagnosis prediction. *NAR Genomics and Bioinformatics*, **6**, lqae022.

Xiao,Y. *et al.* (2018) A deep learning-based multi-model ensemble method for cancer prediction. *Computer Methods and Programs in Biomedicine*, **153**, 1–9.

Xiao,Y. *et al.* (2021) Cancer diagnosis using generative adversarial networks based on deep learning from imbalanced data. *Computers in Biology and Medicine*, **135**, 104540.

Supplementary Table S3. Pairwise distance between cancer and healthy samples with different embedding space.

| Cancer Type | Embedding | Pairwise Distance | | |
| --- | --- | --- | --- | --- |
|  |  | Mean | Minimum | Maximum |
| Lung | mRNA-exmiR | 2.50 | 0.56 | 19.30 |
|  | mRNA-mRNA | 0.36 | 0.05 | 2.22 |
|  | exmiR-exmiR | 0.29 | 0.01 | 0.72 |
| Breast | mRNA-exmiR | 0.35 | 0.10 | 0.85 |
|  | mRNA-mRNA | 0.33 | 0.17 | 0.62 |
|  | exmiR-exmiR | 0.25 | 0.08 | 0.70 |
| Gastric | mRNA-exmiR | 0.33 | 0.01 | 0.86 |
|  | mRNA-mRNA | 0.33 | 0.04 | 0.63 |
|  | exmiR-exmiR | 0.22 | 0.02 | 0.77 |

Supplementary Table S4.

| Cancer Type | Top 5 exmiRs | Targets from full list | Targets from top 100 |
| --- | --- | --- | --- |
| Lung | hsa-miR-4787-5p | KLHL6, TMBIM4, MAPK8IP3, RAVER1  NGFR, FAS, NPR1, HNRNPUL2  CBX7, ATP1B2, PLIN4, HMBOX1  MCAM, GADD45B, KCNJ12, TRAF1  ADM, LYL1, PNPLA7, SRGAP2  STK10, DMWD, MAF, C7  PHACTR1, NPDC1, LIMS2, MCOLN1  RADIL, WTIP, GFRA1, RGS11  CCM2L, RHOC, LDB2, TMEM259  BCO2, SYDE1, LHX6, HES4  CCNL2, EFNB1, NOTCH4, GRK5  PDE8A, NLRP1, LRCH4, CHD2  SHANK3, MYH10, LZTS1, LY6G5B  ARRDC2, GBGT1, TNNC1, FABP4  BEGAIN, SPECC1L, PKD1, OSR1  ELL, CORO2B, WBP1, ABLIM3  AKNA, UAP1L1, LMCD1, MAP4K2  DNAJB5, ALDOA, FZD4, SCARF1  MEFV, DOT1L, AFF2, SORBS3  LRRC4B, MXD1, PGR, USHBP1  BBS5, ARHGEF1, RASA4, PRELP  LCAT, ANKDD1A, MPZ, RBM33  SLC11A1, ZNF580, SDHAF2, RBMS3  JUNB, LIMK2, USP6, SOD3  MYL9, JUND, SLC6A1, PNPLA6  F10, NLGN2, MSX1, NKD1  HMCN2, STRADA, C1QTNF1, HSPB6  YPEL3, CHRD, KIF21B, KLRD1  DMPK, SOX5, GABBR1, CACNA1C  GDPD5, TUB, MAP1LC3C, TSPAN9  ZYX, KIAA0040, EBF2, KCNK3  VN1R1, IQSEC3, RASSF2, SERPINE1  MYLK, C5AR2, RIMS3, HOMER3  RHOB, FAM43A, ENGASE, INPP5E  EOMES, TMEM119, PALM, MDGA1  PTPRS, TSPYL2, FAM107A, CTIF  GPIHBP1, CSNK1G2, SIN3B, TNFAIP2  FLNC, LAMC3, PLCG1, MEGF6  KLHL3, BVES, FLT1, CPLX1  ITIH5, ASGR2, PHOSPHO1 | ANKRD33B, ADM, TMEM259  NLRP1, ABL1, RADIL  SCARF1, LCAT |
|  | hsa-miR-8069 | KLHL6, TSPAN32, RAD50, ETS2  SOCS2, GLI4, PAG1, SERPINB9  DCUN1D3, CREB5, STARD4, ADAMTSL3  BTAF1, KCTD16, EDN1, COL27A1  ESAM, TFEC, STXBP6, EBLN2  DUSP2, CELF2, SHROOM4, RFX3  SLC6A16, PTPRD, ANO2, BCO2  ATXN3, LHX6, NPR3, GPC3  TPST2, ARHGAP10, LDB3, TCEAL4  MPP3, SLC12A4, PRDM5, TUBB1  ARHGAP29, HLA-E, TPCN2, UBE2V1  GPR135, JDP2, TBC1D1, CXCL12  CSF1, COLQ, PRELP, RBM33  ZNF841, BACH2, SLC6A1, ITPKB  SYNE1, GPRIN3, STRADA, PRICKLE2  RAPGEF4, ABCA9, NKD2, CALCRL  FUT1, ZZEF1, TMEM170B, TG  IQSEC3, RASSF2, MAN1C1, ERG  PCSK5, INPP5E, FRMD3, BMPER  UBE2F, GJC1, SH3BP2, EDA  GALNT15, FMO2, GLDN, CLEC1A  PLCB4, TMBIM4, TBX21, GRAP2  ADAT2, TLN1, PARVG, CTNND1  LTV1, SLIT2, APBB2, LEPR  ABLIM2, SOCS3, ARHGEF6, PCF11  KLF7, PREX2, FHL1, IL7R  DAB2, LYVE1, EFHC1, HDAC7  RHOJ, PPARGC1B, POLD4, FOSL2  ELN, UNC5C, SULT1C4, ADSL  ACACB, TMEM86B, ITK, FAM200B  MOB3C, AFF3, SIRPB2, ANKRD36  TNFSF14, HSD11B1, FSTL1, SEMA6D  ANGPT1, MAFG, FZD4, SSH2  RFX2, FERMT2, ZNF423, PGR  LIPG, EMP1, PRKCB, ZNF23  ITGA8, RAPGEF3, CYYR1, FAM219A  SOD2, LRP11, RAB8B, IL1R1  FMOD, TRPC4, LYST, HEY2  LILRA1, COLGALT2, CACNA1C, PEAK1  NID1, TNFAIP3, ROBO2, NAALAD2  RIMS3, FRMD4A, ENPEP, ZNF223  PDE3B, UQCR11, NFASC, USPL1  KCNB1, ARHGAP33, SOBP, ZNF280D  CSGALNACT2, ITIH5, CAV1, ZNF589  DDR2, MYCT1, FLI1, C7  MAL, NMUR1, MXRA7, IP6K3  ASAP1, C1QTNF2, GFPT2, ADAMTS4  TMEM47, PHEX, PTGIS, PRX  PRKX, CRLS1, CLCN6, PDLIM3  SLIT3, MCC, ARRDC2, NEK3  THEMIS2, MAST3, NTRK2, WNT2B  MAP4K2, ATF3, SMAD6, AFF2  C1orf115, SYPL2, LUC7L2, FHL5  CACNA1D, MXD1, TACC1, PIWIL4  IRAK3, ST8SIA1, DCAF8, P2RX1  PARVB, VGLL3, MME, GAS7  GIMAP6, DOK6, RBMS2, GDPD5  ADAMTS17, VN1R1, CCBE1, PSMD9  PDE8B, CAB39L, NCR3LG1, PTPRS  HECW2, DBNDD2, ZNF385D, MCF2L  CD300LB, ALG9, SLFN12L, PRRT1  NXPH3, ZDHHC14, RPP14, CFD  UACA, PPP3CC, GSTM5, CYTIP  TATDN2, LRP2BP, SGIP1, FREM1  UCP3, GFRA1, CDH5, CCDC68  FAM124A, RORA, TNFAIP8L3, ARHGEF10  LPL, CSRP1, GRK5, SLC24A4  APBB1, PLCE1, NFATC1, CNR1  VAMP1, LMCD1, NPNT, CACNA2D3  FGD3, FLCN, DDX17, NABP1  HEYL, C8orf58, HEG1, CLEC2D  MTHFS, NOL12, RBMS3, SMAD9  GUCY1A2, AMOTL1, F10, NEGR1  PEG3, THBS1, PMP22, DST  STX16, CD36, TSC1, TBX5  ADAM33, USP12, SYNPO, CRISPLD2  DKK3, FPR1, CX3CL1 | CDH5, C8orf58, RAD50  KLB, CALCRL, MOB3B  JDP2, PSMD9, MTHFS  PDE3B, FMO2, PRX  PRUNE2 |
|  | hsa-miR-3665 | KLHL6, FOXO3, MAPK8IP3, PCDHGC3  RAD50, HNRNPUL2, RUSC2, PDE2A  NXF1, GLI4, RALGDS, SERPINB9  NLGN3, STARD4, WSB1, SNX15  ADAMTSL3, FKBP1B, TOX2, KCTD16  ARID3B, COL27A1, STXBP6, KCNQ4  PDLIM7, DUSP2, CELF2, RADIL  CCM2L, TMOD2, MT1E, SHROOM4  ATXN3, HES4, CNTNAP1, MPRIP  IGSF10, ARHGAP10, SLC25A37, LUC7L  PIK3R3, SEMA6C, PIEZO2, LDB3  AQP1, MS4A14, LBH, ICA1L  SLC12A4, CD93, SPECC1L, ZHX3  SMTN, KCNC4, CD274, NHSL2  TBC1D2B, TMEM204, MRGPRF, SCARF1  TPCN2, KIF17, CROCC, SVEP1  LZTR1, JDP2, ZNF697, KIF26A  EPOR, SRSF1, RAPGEF5, CDKN1A  CSF1, COLQ, PRELP, ZBTB47  AOC2, LIN7A, NDUFC2, KLHL29  ZDHHC11B, ITPKB, TBC1D20, SRGN  GPRIN3, NKD1, HMCN2, PRICKLE2  KCNMB1, RAPGEF4, ABCA9, TBXA2R  SPOCD1, FUT1, ZNF471, PSTPIP2  CAPN3, TPM2, F8, TSPAN9  EBF2, SPOCK2, TMEM255B, TG  RASSF2, DNAJB4, COL6A1, ZNF284  ACE, ERG, BNC2, PCBP4  RAPGEF1, PALD1, UBE2F, PALM  MDGA1, APOL3, ITGA2B, CCPG1  GJC1, SH3BP2, MRPL12, GALNT15  PRDM6, NUMBL, ZMYM6, MTG1  LTBP4, CD247, GLDN, MYH11  PPM1F, VSTM4, CBFA2T3, NOSTRIN  NGFR, GRAP2, PHYHIP, KCNIP2  CBX7, SH2D3C, ARHGEF26, ATP1B2  PLIN4, SNCA, PARVG, SLCO2A1  TRAF1, SLIT2, APBB2, SRGAP2  NKTR, ABLIM2, F2RL3, CRIM1  ARHGEF6, PDE4B, KLF7, CCDC50  NPDC1, MAMLD1, IL7R, BSCL2  SETD5, DAB2, WTIP, LYVE1  HDAC7, PPARGC1B, POLD4, FADS3  FOSL2, GALNT16, NCF1, UNC5C  ACACB, ZNF25, CCNL2, DKK2  PLAC9, LRCH4, TCF21, NPEPL1  MOCS1, WHAMM, FKBP5, TRPV2  GPR176, EBF4, TDRD10, LY6G5B  SERPING1, SEMA3G, RAB43, FGF2  CD34, AFF3, EML1, ADARB1  COL13A1, SIRPB2, N4BP3, SEMA6D  CYGB, FZD4, JPH2, DNAJB1  POPDC2, ZNF423, LRRC4B, ARHGEF17  NAA10, LIPG, IL15RA, PRKCB  ITGA8, MYSM1, ANKDD1A, ADAM19  DGKD, PAGR1, NR4A3, FAM219A  SOD2, SEC14L1, NOVA2, IL1R1  CLN8, ATP8B2, FMOD, ZNF366  ITGA5, PCDHGB7, LILRA1, LENG8  SUPT4H1, COLGALT2, MYL3, C11orf21  NR5A2, KLRD1, MSRB3, NAV3  IFITM10, FRAS1, PSTPIP1, CACNA1C  CSRNP1, ZYX, USP35, INPP5A  IRAK2, AXIN2, RIMS3, C5AR1  PHLDB1, MICAL3, FRMD4A, TNFRSF14  TMEM119, DCLK2, PDE3B, UQCR11  NFASC, FAM107A, TXK, ID1  USPL1, KCNB1, ASXL1, ULBP1  MEDAG, ZNF280D, CSGALNACT2, P2RX7  RNF166, ITIH5, MAF, ZNF589  R3HDM2, PLIN5, DDR2, ZGPAT  MAP3K3, IL2RG, PITPNM2, GIMAP8  GNG11, HSPA12B, DUSP8, CD300E  INMT, NFATC4, ETS1, DMWD  IL4R, MXRA7, DGKG, ADAMTS2  SLC25A34, ASAP1, TAL1, NR4A1  ZNF331, LMOD1, DOCK6, TCIRG1  PRR16, ASB2, PTGIS, PRX  ABCF2, NLRP1, CLCN6, DUSP1  LZTS1, PKNOX2, ASNSD1, MYOM2  ARRDC2, CCND2, PKD1, MASP1  SAA1, SPNS1, NTRK2, WNT2B  ABLIM3, TRIM66, RUNX1T1, TMEM44  SGCD, MAP4K2, DNAJB5, C2orf88  ALDOA, AP3S2, SMAD6, AFF2  CORO7, ADAMTS8, MKNK1, TEK  LUC7L2, FHL5, PLXNA2, BBS5  IRAK3, SLCO4A1, PXK, EPB41L3  MYLK4, ST8SIA1, CLIP2, SGCA  MPZ, TECTA, P2RX1, NOTCH1  PARVB, SDHAF2, MYL9, KLF2  WAS, CLSTN2, SSH1, CTC1  PLSCR4, GAS7, SRGAP1, CHRDL1  TTC28, SAMD4A, DOK6, YPEL3  GALNT18, MIB2, SRSF11, TUBGCP6  RBMS2, GDPD5, PCDHGA12, S100A8  TUB, ADAMTS17, KIAA0040, SYNGR1  KCNK3, MAD1L1, CCBE1, ARHGEF7  TINAGL1, NR2F1, MTCP1, ZDHHC11  TSHZ3, PDE8B, RHOB, FAM118A  NCR3LG1, ENGASE, AGO4, DBNDD2  CR1, MYO1F, MCF2L, RNF152  DGKH, SIN3B, FLNC, LAMC3  NDUFA9, SH3RF3, C18orf32, ADCY5  LSS, CD300LB, RAB5C, SOX18  ASGR2, C14orf132, SRL, ALG9  SLFN12L, FGFR1, NXPH3, JAK3  ZDHHC14, NPR1, CDH13, ZBTB43  ISY1, GAB3, SPICE1, CFD  SLC35D2, OTUD7A, PNPLA7, PHLDB2  MYRF, GSTM5, CYTIP, MAMDC4  TATDN2, TEAD4, PIM1, PLEKHG1  UCP3, GFRA1, NTNG2, RGS11  LILRA6, LIF, PDLIM2, FAM124A  CALCOCO1, FLNA, TNFRSF1B, TNFAIP8L3  AGAP9, PPP1R12B, KLF6, CTTNBP2  FCN1, EVI2B, ADAMTS13, S1PR1  CSRP1, C1QTNF7, SLC24A4, CHD2  APBB1, CDC37, IFFO1, DAB2IP  NFATC1, BCAP29, GBGT1, SPNS2  LRRC32, GIGYF1, FOXF2, SOX13  ARSI, NRBP2, SEMA6A, AKNA  PRG4, LMCD1, SLC25A32, TNS1  HIF3A, FAM111A, SNTB2, MYO9B  DDX17, GHR, NABP1, HEYL  GTF2IRD2B, CAMK2N1, GPER1, MEIS1  MTHFS, TAP2, BRICD5, DLG4  NOL12, RNF217, PGAP1, TMEM88  SLC11A1, SMAD9, RHBDF1, MFNG  AMOTL1, F10, NEGR1, PPP1R15A  PPP1R18, C16orf86, TTC7B, KIF21B  THBS1, CXCR2, RASL12, SH2B3  DST, ATG9A, PEAR1, SERPINE1  MYLK, SMIM10, CD36, FMO3  KCTD15, STAT5B, SCN4B, C3orf62  ADAM33, WDFY2, SLC1A3, S1PR2  SYNPO, CRISPLD2, GPIHBP1, LAMA4  ZNF449, RASD2, SLC2A6, BVES  SOX17, STARD8, DKK3, FBF1  FPR1, CX3CL1 | RAD50, NLRP1, MOB3B  ABL1, DUSP1, MTHFS  MYSM1, BCAP29, AQP1  ANKRD33B, TFPI, MECOM  PDE3B, SPNS1, CCPG1  BOK, RADIL, SNED1  SCARF1, JDP2, GHR  PRX |
|  | hsa-miR-4488 | RAD50, MAPK8IP3, PDZD4, FBXL7  NGFR, MT2A, CBFA2T3, DNLZ  CD248, RAVER1, KCNIP2, ADAT2  IL2RG, ARHGAP23, ATP1B2, GLI4  PARVG, HMBOX1, LAMB2, SDHA  ABTB1, SLCO2A1, CFD, EDC4  DUSP8, NFATC4, CRELD1, TRABD2B  MAF, DMWD, RIN3, VAMP2  KLF7, PHACTR1, ADAMTSL2, KCNQ4  KCND1, DGKG, UCP3, RADIL  ARPC1B, HDAC7, LILRA6, TMEM259  FLNA, TCIRG1, CLDN5, ACACB  PRX, ZFP36L2, FCN1, EFNB1  ADAMTS13, TMEM86B, CHTF8, NLRP1  LRCH4, LILRB2, ITGAX, SHANK3  LZTS1, PKNOX2, LILRB5, EBF4  ICAM5, BEGAIN, PREX1, OSCAR  SPNS2, RAB43, SLC12A4, SPECC1L  KAT2A, OSR1, STC2, MAST3  COL13A1, SPI1, TNFSF14, GPR3  MASP1, KAZN, NTRK2, COL6A6  WNT2B, HLA-E, FSTL1, NHSL2  N4BP3, ABHD17A, TNS1, MAP4K2  REM2, GRID1, SBNO2, SCARF1  TPCN2, MYO9B, MKNK1, SORBS3  JDP2, FHL5, TBKBP1, HEYL  PLXNA2, JPH4, CLEC2D, COLQ  EPAS1, CLIP2, RASA4, ZCCHC24  DLG4, CLEC3B, DGKD, CEBPB  FAM219A, SOD2, JUNB, C17orf49  TAGLN, NDRG4, ACVRL1, KLHL17  SEC14L1, KLF2, CLSTN2, POU6F1  GIPC3, NEGR1, SETBP1, GAS7  JAM2, ITGA10, GPRIN3, CDH6  NKD1, HEY2, EGFL7, KCNMB1  C1QTNF1, LENG8, C16orf86, YPEL3  MIB2, CEACAM19, TUBGCP6, DMPK  PSTPIP1, KIAA0040, S1PR4, KCNK3  TG, SERPINE1, LRP1, TINAGL1  TNFSF12, SYNGAP1, TTLL3, NCKAP5L  PCBP4, RHOB, GPSM1, FAM43A  SNAI1, HPD, LILRB3, PALM  PTPRS, PTGIR, ULBP1, MCF2L  ARHGAP33, ADAMTS7, HSPB7, CENPT  IL1RL1, CSNK1G2, CRISPLD2, WDR45  SIN3B, BCL6B, SYNM, FOXO1  SOX7, FAM167B, CPLX1, MYH11  ASPA, PPM1F, CCDC88B, CX3CL1 | ANKRD33B, RAD50, EDC4  MT2A, TMEM259, NLRP1  BOK, JDP2, RADIL  LRP1, SCARF1, PRX |
|  | hsa-miR-3960 | MAPK8IP3, PDZD4, CBFA2T3, QRICH2  CXCL12, PLIN4, MAN1C1, CFD  DGKD, DUSP8, MICAL3, TSFM  SLC11A1, RAB43, RBPMS2, PAM16  PKD1, SLC6A1, ATN1, TSPYL2  ADAMTSL2, FMNL1, ALKBH6, CTC1  ZNF467, ADAMTS10, SLC25A29, C11orf96  SLC25A34, NDUFA11, STRADA, ABHD17A  EHBP1L1, CCM2L, LENG8, DOT1L  MMP25, LTBP4, MIB2, MYO9B  CPLX1, DMPK, EMILIN1, KANK2  LRRC4B, ASGR2, PHOSPHO1, JAG1  PRX | PRX |
| Breast | hsa-miR-3960 | SLC29A4, MAPK8IP3, PDZD4, CD209  CXCL12, CACNA1A, PLIN4, MAFK  JAG1, CFD, OSR2, HOXD3  MICAL3, TSFM, SLC11A1, RAB43  RBPMS2, ARHGEF4, RBM25, PAM16  PKD1, PLVAP, PIM3, SLC6A1  ATN1, WDR91, TSPYL2, ALKBH6  USP2, NAT8L, ADAMTS10, ABHD17A  C11orf96, SLC25A34, NDUFA11, STRADA  EHBP1L1, HOXA9, CCM2L, WSCD1  NMNAT2, LENG8, LTBP4, MIB2  ZFP36L1, CPLX1, DMPK, ZDHHC8  KANK2, LIPE, LRRC4B, EMILIN2  PRX | LENG8, ZFP36L1, C11orf96, PLIN4, PIM3, CFD |
|  | hsa-miR-4488 | ANKS6, RAD50, MAPK8IP3, PDZD4  MT2A, NGFR, DNLZ, CD248  RAVER1, ADAT2, KCNIP2, FASN  ARHGAP23, ATP1B2, GLI4, TBX18  SDHA, ABTB1, SLCO2A1, CFD  EDC4, NFATC4, CRELD1, CTDNEP1  TRABD2B, MAF, DMWD, VAMP2  KLF7, ADRA2A, KCNQ4, KCND1  DGKG, BOK, RADIL, ARPC1B  WSCD1, HDAC7, LILRA6, TMEM259  ACSL4, TCIRG1, CLDN5, ACACB  DNM1, PRX, ZFP36L2, GK5  EFNB1, TMEM86B, NLRP1, LRCH4  SHANK3, LILRB5, PDE7B, BEGAIN  SPNS2, RAB43, GRM2, SLC12A4  KAT2A, OSR1, TNFSF14, GPR3  COL6A6, PPP1R1A, ACBD4, HLA-E  FSTL1, NAT8L, TNS1, ABHD17A  SLC39A14, GRID1, SBNO2, SCARF1  TCN2, AFF1, TPCN2, MKNK1  SORBS3, JDP2, FHL5, PID1  TBKBP1, PLA2G6, PLXNA2, RASD1  SLC29A4, RAB40A, JPH4, CES4A  GSC, VEGFB, ATOH8, EPAS1  RASA4, ZCCHC24, ANKRD33B, CLEC3B  CEBPB, SCN1B, INF2, FAM219A  SOD2, JUNB, C17orf49, ADAM11  NDRG4, ACVRL1, KLHL17, SEC14L1  KLF2, POU6F1, RERE, JAM2  ITGA10, CDH6, NKD1, EGFL7  PLEKHG5, KCNMB1, C1QTNF1, LENG8  HDAC10, YPEL3, C16orf86, RGMA  MIB2, CEACAM19, ZFP36L1, TUBGCP6  DMPK, DPYSL2, TFCP2L1, EGFLAM  HEBP2, ELMOD3, TG, PTP4A3  MAPRE3, STXBP1, CACNA1A, SERPINE1  LRP1, TINAGL1, TNFSF12, ELFN1  HLF, PFKFB3, TTLL3, RHOB  FAM43A, SNAI1, PMF1, PALM  RHOU, PTPRS, RASL10B, C1orf50  PTGIR, MCF2L, ARHGAP33, ADAMTS7  HSPB7, CENPT, IGF2BP2, CRISPLD2  ADCYAP1R1, WDR45, SIN3B, BCL6B  FOXO1, SOX7, NPTX2, CPLX1  MYH11, PPM1F, CX3CL1 | LENG8, MT2A, CD248  HLA-E, CEBPB, KLF2  RHOB, JUNB, ZFP36L1  FASN, CLDN5, CFD  RASD1 |
|  | hsa-miR-3665 | TLN2, RAD50, MAPK8IP3, HNRNPUL2  RUSC2, PDE2A, NXF1, GLI4  RALGDS, WSB1, SNX15, LYG1  FKBP1B, TOX2, COL27A1, STXBP6  KCNQ4, BOK, CELF2, RADIL  CCM2L, WSCD1, MT1E, SHROOM4  ATXN3, HES4, MUC3A, CNTNAP1  MPRIP, MAP1LC3B, IGSF10, ARHGAP10  SLC25A37, LUC7L, SBF1, SCN4A  SEMA6C, ECHDC3, LDB3, AQP1  TFPI, ICA1L, SLC12A4, PLIN1  DNAJC27, CD93, ZHX3, SMTN  CACHD1, KCNC4, TBC1D2B, TMEM204  PCBP3, MRGPRF, SCARF1, SLC25A10  TPCN2, KIF17, CROCC, SVEP1  LZTR1, JDP2, FAHD2A, SLC29A4  KIF26A, EPOR, SRSF1, RAPGEF5  CDKN1A, CSF1, TLR4, PRELP  ZBTB47, VEGFA, CHST2, ANKRD33B  BMP2, AOC2, NDUFC2, TLCD2  KLHL29, GTF2IRD2, SLC16A7, MAML2  SRGN, NIPSNAP3B, NKD1, HMCN2  KCNMB1, ZBTB16, ABCA9, ZNF471  MITF, ZDHHC8, CAPN3, TPM2  MATN2, F8, TFCP2L1, FGFRL1  EBF2, TMEM255B, TG, FBLN2  RRAGC, GNAI1, ERG, KSR1  ZFYVE28, RASSF4, UBE2F, PALM  RASL10B, APOL3, FAM124B, CCPG1  SH3BP2, MRPL12, GALNT15, NUMBL  ZMYM6, MTG1, EGFR, LTBP4  GLDN, MYH11, PPM1F, PRR5  VSTM4, ANKS6, NOSTRIN, NGFR  NOD1, PARM1, KCNIP2, PHYHIP  CBX7, XG, SH2D3C, ATP1B2  PLIN4, SLCO2A1, TRAF1, TTC38  NKTR, ABLIM2, PTBP2, F2RL3  CRIM1, KLF7, CCDC50, NPDC1  MAMLD1, BSCL2, DAB2, CPM  NR3C2, WTIP, LYVE1, HDAC7  AIFM2, MAP1B, IL33, PPARGC1B  POLD4, LPIN3, FADS3, DOC2B  FOSL2, ACACB, PITPNC1, SHC3  CCNL2, PLAC9, LRCH4, NPEPL1  MOCS1, WHAMM, FKBP5, TDRD10  LY6G5B, MECOM, SERPING1, SEMA3G  RAB43, FGF2, CD34, KTI12  EML1, CYGB, INHBB, PELI2  FZD4, JPH2, ZNF300, POPDC2  ZNF423, LRRC4B, ARHGEF17, NAA10  IL15RA, PLA2R1, FBXO31, VEGFB  CALB2, ITGA8, MYSM1, THRSP  ANKDD1A, RPS6KA2, NR4A3, SOD2  FAM219A, ARHGEF4, PLCH2, SEC14L1  NOVA2, IL1R1, C19orf12, ZNF366  ITGA5, GPR35, PCDHGB7, LENG8  NUAK1, SUPT4H1, TSHZ2, MYL3  COX14, OSMR, NR5A2, FMNL2  MSRB3, IFITM10, CSRNP1, QKI  STXBP1, IRAK2, MAFK, AXIN2  PHLDB1, MICAL3, MEOX1, TNFRSF14  DCLK2, PDE3B, UQCR11, FAM107A  ID1, KCNB1, ASXL1, CFL2  MEDAG, ZNF280D, NPTX2, FBXO44  ITIH5, MAF, CYB5R3, R3HDM2  PLIN5, DDR2, ZGPAT, MAP3K3  RPS6KA3, PITPNM2, GIMAP8, PRDM16  GNG11, LPIN1, HSPA12B, INMT  NFATC4, ETS1, CAMK1, DMWD  SV2B, MXRA7, DGKG, SLC25A34  FAM89A, TAL1, NR4A1, ZNF331  CDC14B, RSPO3, LMOD1, F13A1  DOCK6, TCIRG1, PRR16, KCTD12  PTGIS, DNM1, PRX, ABCF2  NLRP1, CLCN6, DUSP1, PIK3IP1  ASNSD1, LRIG3, ARRDC2, STAB2  PKD1, PAOX, SAA1, JAG2  SPNS1, ABLIM3, TRIM66, RUNX1T1  MEF2C, DNAJB5, C2orf88, PLEKHF1  PRDM8, ALDOA, AP3S2, SMAD6  CYTH3, LAMC1, CORO7, MKNK1  TEK, LUC7L2, FHL5, PLXNA2  RASD1, BBS5, RAB40A, IRAK3  SLCO4A1, EPB41L3, MYLK4, MPZ  TECTA, INF2, NOTCH1, PARVB  SDHAF2, ITGB3, MYL9, NRGN  KLF2, PIM3, PLSCR4, CHRDL1  SAMD4A, SNED1, PCDHGB6, CASP10  YPEL3, MIB2, SRSF11, TUBGCP6  RBMS2, RNF146, DPYSL2, GDPD5  TUB, DUSP22, ANK2, TMEM43  MAPRE3, MOB3B, CCBE1, ARHGEF7  TINAGL1, NR2F1, MTCP1, OSR2  PDE8B, RHOB, FAM118A, ENGASE  SLFN11, SLC9B2, NNMT, DBNDD2  C1orf50, MCF2L, DGKH, SIN3B  FLNC, LAMC3, NDUFA9, SH3RF3  TWIST1, C18orf32, ADCY5, SOX18  RAB5C, SRL, ALG9, FGFR1  NPR1, THSD7A, ISY1, ABL1  GAB3, SPICE1, SRSF4, CFD  SLC35D2, OTUD7A, PNPLA7, PHLDB2  FOXP2, GSTM5, MAMDC4, LPAR1  TATDN2, PIM1, BHMT2, PLEKHG1  LILRA6, PDLIM2, FAM124A, CALCOCO1  TNFRSF1B, AGAP9, PPP1R12B, KLF6  CTTNBP2, DMGDH, S1PR1, IL20RB  C11orf54, APBB1, CDC37, CHST3  IFFO1, DAB2IP, KLF8, BCAP29  GBGT1, SPNS2, LRRC32, GIGYF1  RPL13, IRF4, NRBP2, SEMA6A  AKNA, LMCD1, NAT8L, SLC25A32  TNS1, SNTB2, TCN2, GHR  NABP1, RASGRF2, GTF2IRD2B, SGSM2  GPER1, CES4A, ZFAND5, ALPK3  ZNF334, MEIS1, MTHFS, TAP2  CCDC14, NOL12, RNF217, ANTXR2  TMEM88, SLC11A1, SMAD9, MFNG  GLYCTK, F10, IRS2, LDHD  PPP1R15A, PLEKHG5, GLUL, NMNAT2  C16orf86, RGMA, TTC7B, KIFC3  EPS8, LIPE, RASL12, COL28A1  ANGPT2, DST, ATG9A, ELMOD3  PEAR1, SERPINE1, SMIM10, CD36  FMO3, TMEM220, STAT5B, CLTCL1  SCN4B, C3orf62, ADAM33, WDFY2  SLC1A3, SYNPO, GPIHBP1, CRISPLD2  ADCYAP1R1, LAMA4, ZNF449, SOX17  STARD8, FBF1, OAF, PML  FNTB, CX3CL1 | S1PR1, DUSP1, PLIN4  THRSP, CFD, AQP1  RHOB, SERPING1, PLIN1  RPL13, F2RL3, KLF2  PIM3, SAA1, ID1  PPP1R15A, GLUL, ALDOA  MT1E, LENG8, TPM2  SOX18, RASD1 |
|  | hsa-miR-6869-5p | BDH2, FGFR1, ARHGAP26, TRPC6  RAD50, NPR1, ETS2, MYCT1  STOX2, PARM1, XG, RPS6KA3  RPP14, GNG11, MAN2A2, DOCK11  CFD, WASF3, CREB5, TRAF1  GIPC2, PKD2, MCTP1, TRIOBP  SPRY1, CTDNEP1, SOCS3, PTBP2  SLC35A1, F2RL3, BTAF1, FOXP2  ZMAT1, IL18R1, PDLIM1, VAMP2  FHL1, TCF7L2, ESAM, KLF4  CSNK1A1, STXBP6, CAT, MXRA7  DAB2, CPM, FIGN, FAT4  PDE3A, ELMO1, PTPRB, SHROOM4  MAP1B, RHOJ, IL33, POLD4  PPARGC1B, ADAMTS4, MSANTD2, EMCN  FOSL2, PPARG, NBPF12, ACSL4  FAM124A, TLL1, TNFRSF1B, RORA  ZDHHC17, TCIRG1, AKR1C2, PRR16  ABCA1, ATXN3, KCTD12, AVPR1A  PPP1R12B, GK5, CCNL2, DMGDH  CBLB, MPRIP, MAP1LC3B, L3HYPDH  SLIT3, FKBP5, FAM200B, ELOVL7  ZNF662, NR2C1, TYRO3, LILRB5  APOL4, ASNSD1, KLF8, LY6G5B  LRIG3, ZNF781, PDE7B, TFPI  SERPING1, ICA1L, DNAJC27, HYPK  RPL13, CD93, ASL, EML1  DAAM2, NAMPT, ZHX3, PAOX  CORO2B, KLHDC1, TNFSF14, GPR3  LMO3, CEBPD, HSD11B1, TANC1  HLA-E, GADD45A, PIWIL2, DNM3  MEF2C, DNAJB5, ATF3, C2orf88  HAND2, SNTB2, CYGB, AMACR  HBEGF, FZD4, ALDOA, PRUNE2  AP3S2, TCN2, COL21A1, CDK15  FERMT2, SYPL2, PDE4D, CORO7  PLEKHG2, TEK, JDP2, FHL5  ZNF423, ST6GALNAC3, GHR, C6orf163  RAPGEF2, SLC2A4, FBXO17, EMP1  TACC1, ABCC9, SGSM2, MEIS2  CYP26B1, PNRC1, RAB40A, BTNL9  EPOR, SRSF1, RAPGEF5, TLR4  FGD4, KRIT1, LATS2, PRELP  TGFBR2, CYYR1, NRP1, LRRC8C  LCAT, SHPK, RNF217, PC  NR4A3, GFOD1, RBMS3, SDHAF2  JUNB, AOC2, AFAP1L1, KBTBD11  AASS, KCNAB1, NDUFC2, TLCD2  HOXD9, F3, LRCH2, VGLL3  ZNF354B, ZC3H12C, MAML2, NACAD  C1S, BST1, NIPSNAP3B, RASL12  ADAMTS5, CDH6, NKD1, SNED1  KCNMB1, CSAD, GLUL, GOLGA8B  ZNF117, UCHL3, NUAK1, TSHZ2  CALCRL, ZNF471, EDNRB, MSRB3  ID4, EPS8, TFCP2L1, CSRNP1  TUB, RIC3, LY75, HEBP2  EMILIN2, ANGPT2, QKI, DST  ZNF19, ADAMTS3, TMEM43, MMRN1  DZIP1L, STX16, MOB3B, CCBE1  GPAM, ITSN1, NAALAD2, AXIN2  ERG, FAM20A, HELB, PDE8B  TSC1, SYNPO2, ABCB1, TBX5  AKAP12, RCAN2, GOLGA8A, UQCR11  SEMA3A, RHOU, NPHP3, NNMT  DBNDD2, AGPAT4, APOL3, WDFY2  ID1, MICA, KCNB1, ABCA5  MMD, NEXN, SH3BP2, IGF2BP2  DGKH, MGAT1, ZNF280D, PAPLN  KCNJ15, ZMYM6, KLB, ITM2A  EGFR, NDN, LTBP4, NPTX2  SOX17, STEAP4, ADCY5, AHR  AK1, ITIH5, MAF, PLAGL1  PML | HLA-E, ID1, SERPING1  SPRY1, JUNB, SOCS3  RPL13, F2RL3, GLUL  ALDOA, CFD, CEBPD |
|  | hsa-miR-6729-5p | PRRT1, TLN2, MAP3K3, C8orf58  MYCT1, MAPRE3, ZFAND5, POLR2M  ZC3H12A, WHAMM, ABL1, SLC25A37  CACNA1A, DUSP1, LUC7L, NRIP2  ITSN1, SCN4A, SHANK3, TMEM91  PEMT, TYRO3, ZNF783, CHST3  NABP1, ANKDD1A, ANKRD33B, ADM  ZFYVE28, PHLDB1, MICAL3, EHD2  SRCAP, RPL13, SMARCE1, DMWD  MMRN2, ACVRL1, PPP1R3G, NRGN  TCF7L1, NOVA2, ISM1, DDX39B  COL27A1, RPP14, TNFRSF10B, VSIG4  LDHD, JAM2, NLGN2, TNS1  C11orf96, SH3BP2, CDH6, GIMAP6  WTIP, PHYHD1, MC1R, LAMC3  RHOJ, KCNJ15, HDAC10, ADAMTS4  DDX19A, IFITM10, LUC7L2, SLC2A4  QKI, EMILIN2 | HLA-E, ID1, SERPING1  SPRY1, JUNB, SOCS3  RPL13, F2RL3, GLUL  ALDOA, CFD, CEBPD |
| Gastric | hsa-miR-4488 | TTLL3, NPTX2, NDRG4, LENG8  KCNMB1, C1orf50, ARHGAP33, ZNF76  SNAI3, ANKRD65, TNS1, TNFSF12  PCBP4, BAIAP2, CDK18, FOSB  RAB11FIP2, DLG4, KCNIP2, LRCH4  WDR45, VAMP2, OSR1, CRELD1  HPD, SLC12A4, ZCCHC24, TG  SCN1B, PTP4A3, NLRP1, JPH4  CLDN5, CRISPLD2, TFCP2L1, SLC7A8  KLHL17, RAD50, KCNQ4, SPRN  GLI4, FAM174B, ABTB1, PMF1  PTPRS, ZNF747, PID1, SH2B1  SDHA, ADAMTS13, SLC25A42, PRX  RASD1, B3GAT1, MBD3, MAPK8IP3  ACACB, CD2BP2, ADAT2, TMEM80  RHOB, EBF4, THTPA, DMPK  ARPC1B, ELMOD3, MT2A, ERN1  DNLZ, C17orf49, ITGA10, RAB43  PHACTR1, GDF7, INPP5J, ADAM11  NUDT16, NANOS1, GPSM1, TUBGCP6  PKNOX2, SPECC1L, COBLL1, CHTF8  SETBP1, CLU, ATP1B2, RGMA  RADIL, HLF, MAPRE3, RASA4  LYRM9, TMEM86B, C16orf86, KCNK3  PTGIR, AVPR2, PLEKHO1, TBKBP1  NEGR1, IL1RL1, NFATC4, CILP  PPP1R1A, CCDC149, HOMER2, KAT2A  NOP2, DGKD, ATOH8, ACBD4  PDZD4, HSPB7, TOM1L2, COL13A1  UBXN6, FBXL7, CFD, NEK9  CLEC3B, CEACAM19, TPCN2, COLQ  CENPT, RAP1GAP2, PIANP, C3orf18  RTEL1, PDE7B, ASPA, UCP3  FHL5, POU6F1 | RASD1, MT2A, LENG8, FOSB, CFD, RHOB, |
|  | hsa-miR-6869-5p | CSRNP1, POFUT2, PTGR2, FZD4  TICAM2, BMPR1B, DZIP1, NPTX2  TRIM9, KCNB1, KCNMB1, C22orf39  CYB5R1, BTNL9, GFOD1, GSTA4  ICA1L, AOC2, SSTR2, TTLL7  BNIP3, HBEGF, NT5DC3, SLC16A10  AASS, TSC1, GHR, PAOX  ZNF285, STK32B, EPN3, TACR2  MSANTD2, AKAP12, ZMYM6, RHOBTB3  ZNF85, AK1, LETMD1, VAMP2  PRELP, PEX6, ATP1A2, ENO2  SGSM2, ZNF471, FAXC, NPHP3  SRSF1, CDHR3, SDHAF2, UBE2G2  TPD52L1, NR4A3, PRUNE2, IL33  LMO3, SYNPO2, ZNF781, ZNF655  CACNB4, MSRB3, GSTM3, ABCC9  AFF3, TFCP2L1, FIGN, RAD50  CCBE1, PPP1R3E, AKR1C2, KLHDC8A  RIC3, N6AMT1, TEF, ZNF280D  KCNT1, PLAGL1, FERMT2, ADH1B  SOCS3, MAGIX, ZNF747, SLC1A2  RNF217, CSAD, STEAP4, WIPF3  DLG2, SSBP2, NAALAD2, NBPF12  XKR4, TMOD2, ARHGAP29, FUT1  HYPK, MICALL1, DDX5, PDE8B  KIAA0513, FBXO9, TSPYL4, TECPR1  ROGDI, TBC1D30, ZNF175, SLC2A11  ATF3, AMACR, PPM1K, ZHX3  GOLGA8B, FBXO17, BSPRY, CERS4  LONRF2, TRAF1, FAM200B, ZNF844  INTU, ZNF726, KRIT1, ADAMTS3  NDUFC2, NANOS1, WASF3, MMRN1  ADAMTS4, ITIH5, POLD4, PAGR1  GLUL, COL21A1, LRRC2, KLHDC1  FHL1, TBCEL, CHTF8, SOX17  JAKMIP2, NEK10, SLC2A4, SETBP1  ZNF284, DMGDH, DIXDC1, BVES  CSNK1A1, AJAP1, GOLGA8A, ASNSD1  TNFAIP8L3, NACAD, LRCH2, SRSF6  ABCA5, L3HYPDH, RPP14, KCNJ15  KCNK3, TUB, LAYN, AVPR2  ZMAT1, SLC33A1, GPM6B, ZNF302  ID4, CDH2, IL18R1, WDFY2  NPR1, STOX2, PTGS1, SYPL2  NEGR1, MTX3, ZKSCAN7, GALNT4  SLC35A1, GCNT4, RCAN2, PDGFD  KIT, REXO2, CORO2B, CORO7  FBXW4, DAAM2, SLC25A4, GPR155  ADCY5, SHPK, ATP8A1, C14orf132  DBNDD2, STX16, LCAT, PKIB  C1S, WDR37, ZFP28, DNAJC27  BTAF1, UQCR11, ZNF404, FGFR1  ZNF662, MYRF, RAB9B, DNAJB5  GPR162, ZDHHC17, IVD, ALDOA  PRR13, CCNT2, PRKAB2, EPOR  FBXL13, CFD, C7, BDH2  ZNF354B, FST, BCL2L2, GADD45A  AP3S2, SCUBE1, FKBP5, PDE7B  CCNL2, REEP1, INSIG1, GDNF  NEXN, NWD1, FHL5, PKD2  FAM20A | CSRNP1, DDX5, GADD45A  ALDOA, SOCS3, CCNL2  INSIG1, CFD, POLD4  GLUL |
|  | hsa-miR-8072 | HSF4, RASD1, PTPRS  LIFR, SYNGR1, SCLY  PRKAG2, GNG7, PRELP  HK3, SLC7A8, SH3GLB2 | RASD1 |
|  | hsa-miR-3960 | PKD1, MICAL3, ALKBH6, LENG8  NANOS1, ATP8A2, PAM16, ING5  STRADA, ADAMTS10, RXRB, SLC25A4  C11orf96, QRICH2, ZNF747, DGKD  RBPMS2, PDZD4, CNTN2, ENDOV  PRX, TSFM, PLIN4, KLHL21  TXNRD2, TSPYL2, SPTBN4, PCGF3  MAPK8IP3, USP2, CFD, CTC1  ZNF784, DMPK, WDR91, KANK2  SORBS2, NTN1, CYTH2, ZNF467  CACNA2D2, NDUFA11, ARHGEF18, CACNA1I  RAB43 | CFD, C11orf96, TSPYL2, LENG8 |
|  | hsa-miR-6729-5p | SLC35F1, ZFYVE28, ARVCF, MICAL3  TMEM91, USP40, LDHD, PHYHD1  ADAMTS4, LUC7L, SPRN, CHST3  C11orf96, MDGA1, NLGN2, TNS1  C8orf58, ZNF747, DGKD, WTIP  SLC2A4, DDX39B, MLPH, ZNF783  ANKDD1A, ADAMTS13, BVES, SRCAP  DDX19A, SMARCE1, MAPRE3, POLI  CLN8, DUSP1, RPP14, KCNJ15  ZNF862, LUC7L2, MC1R, CPE  PRRT1, ADM, PPP2R5B | C11orf96, DUSP1 |


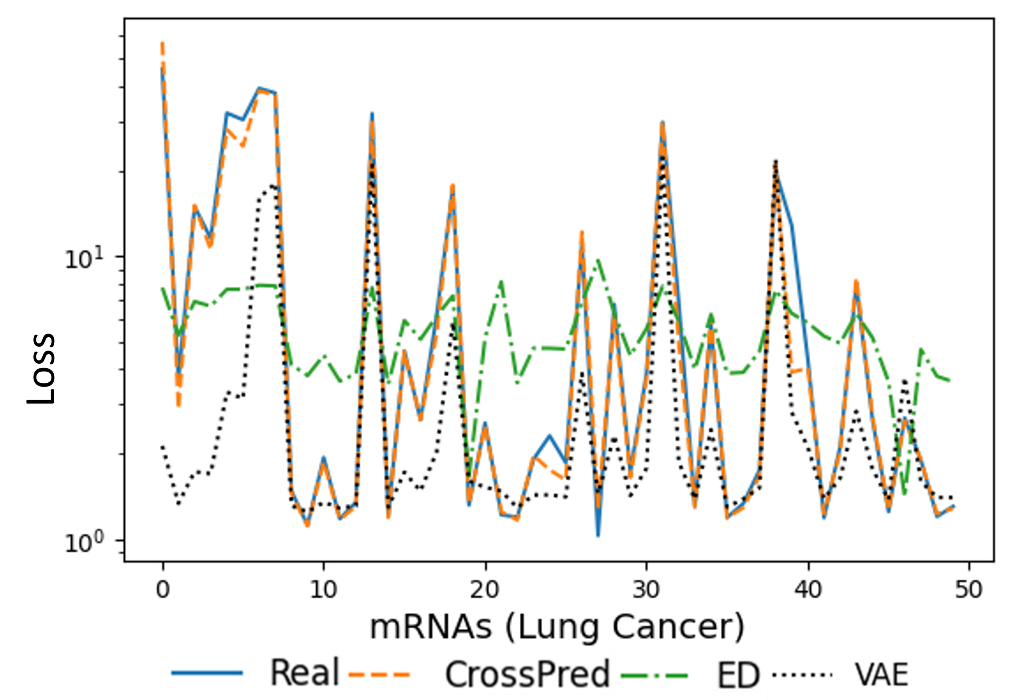


Fig S1. Negative loglikelihood loss of top 50 differentially expressed mRNAs of lung cancer.


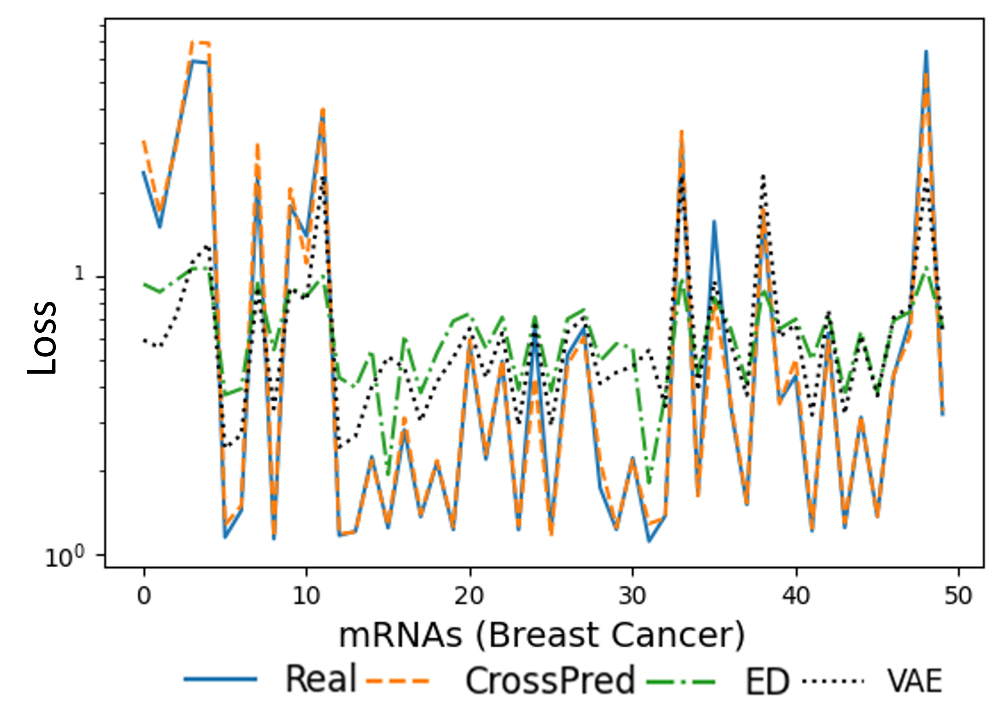


Fig S2. Negative loglikelihood loss of top 50 differentially expressed mRNAs of breast cancer.


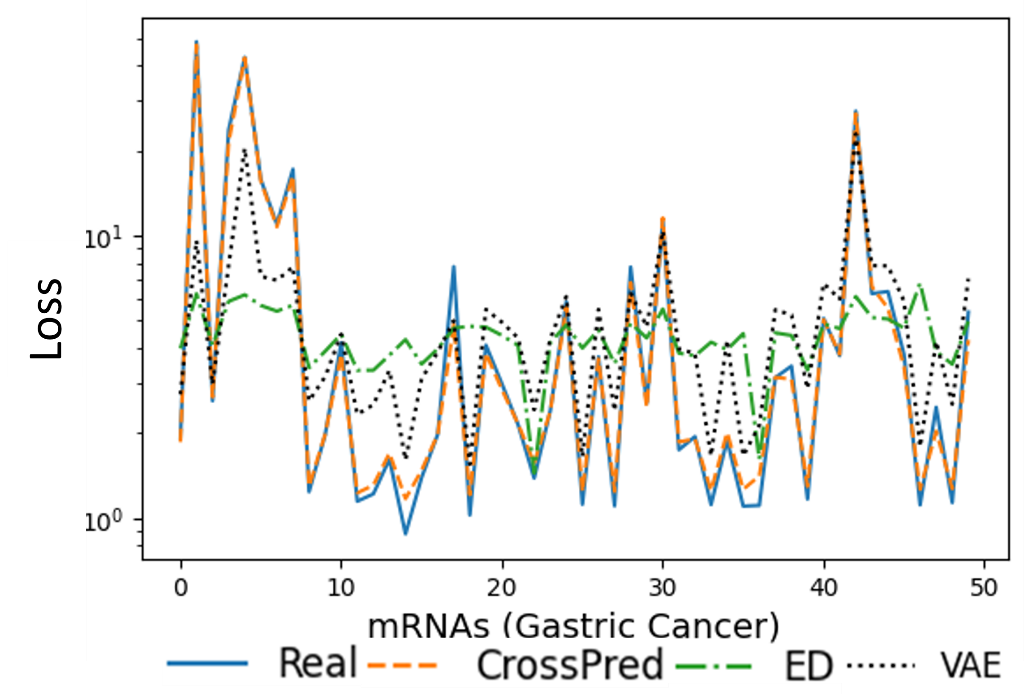


Fig S3. Negative loglikelihood loss of top 50 differentially expressed mRNAs of gastric cancer.


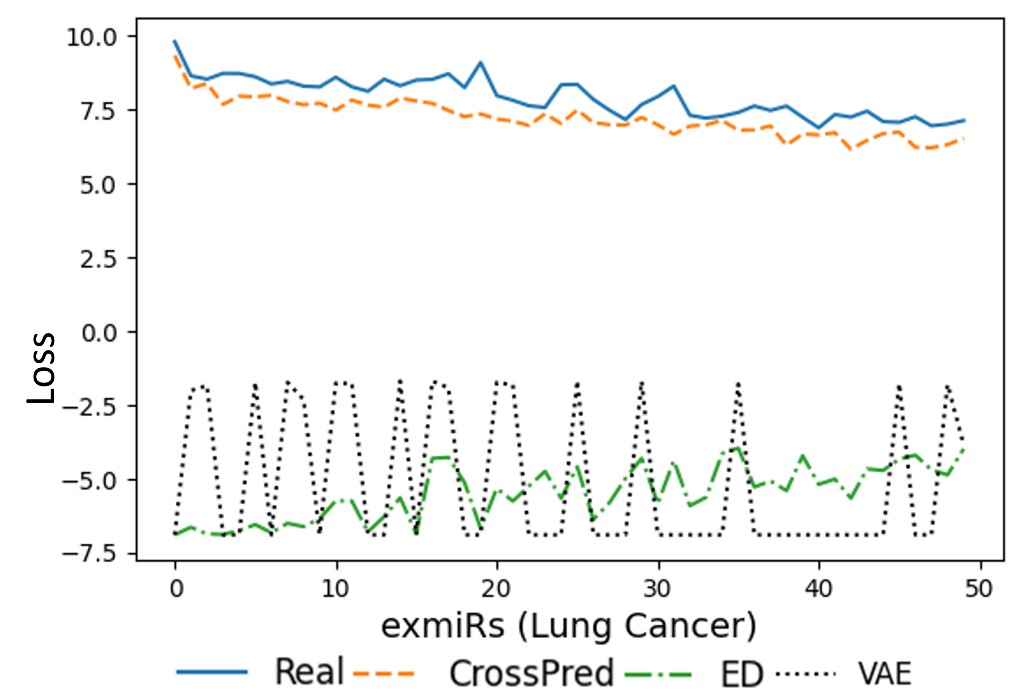


Fig S4. Negative loglikelihood loss of top 50 differentially expressed exmiRs of lung cancer.


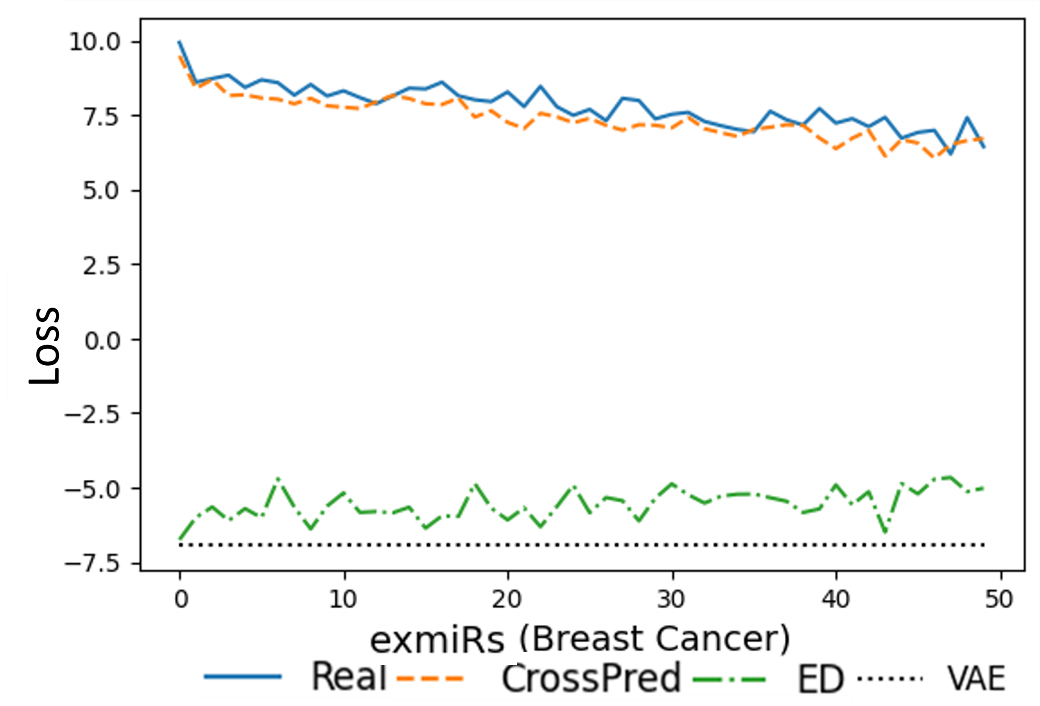


Fig S5. Negative loglikelihood loss of top 50 differentially expressed exmiRs of breast cancer.


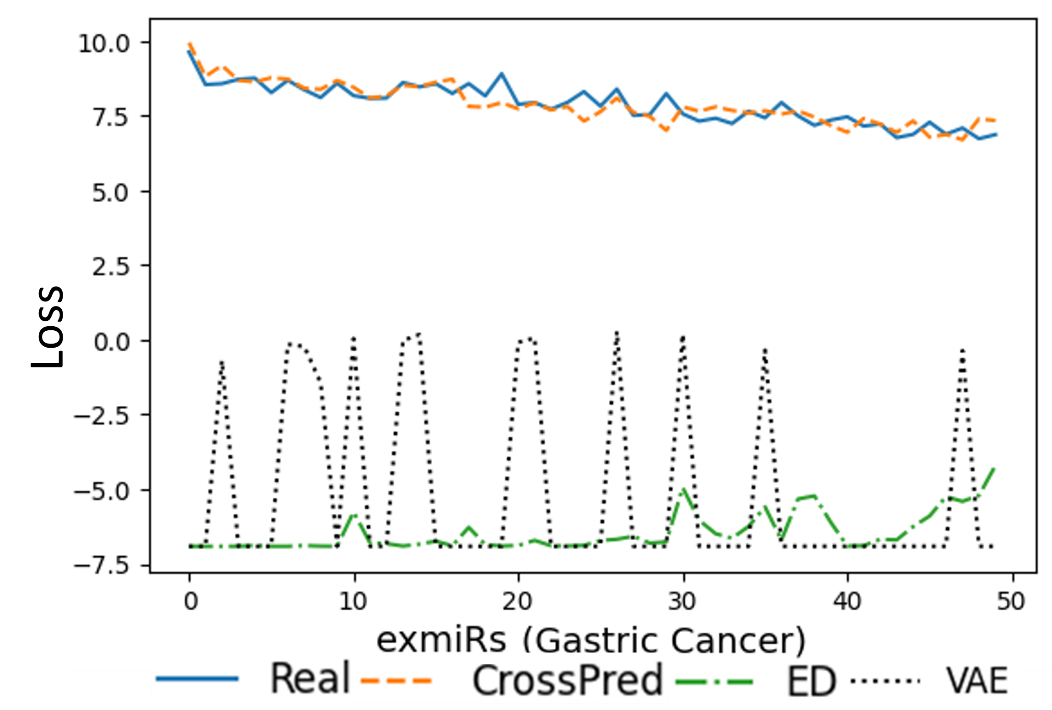


Fig S6. Negative loglikelihood loss of top 50 differentially expressed exmiRs of gastric cancer.
